# Distinct Requirements for Cranial Ectoderm and Mesenchyme-Derived Wnts in Specification and Differentiation of Osteoblast and Dermal Progenitors

**DOI:** 10.1371/journal.pgen.1004152

**Published:** 2014-02-20

**Authors:** L. Henry Goodnough, Gregg J. DiNuoscio, James W. Ferguson, Trevor Williams, Richard A. Lang, Radhika P. Atit

**Affiliations:** 1 Department of Pathology, Case Western Reserve University, Cleveland, Ohio, United States of America; 2 Department of Biology, Case Western Reserve University, Cleveland, Ohio, United States of America; 3 Department of Craniofacial Biology, University of Colorado School of Dental Medicine, Aurora, Colorado, United States of America; 4 Visual Systems Group, Cincinnati Children's Hospital Medical Center, Cincinnati, Ohio, United States of America; 5 Department of Genetics, Case Western Reserve University, Cleveland, Ohio, United States of America; 6 Department of Dermatology Case Western Reserve University, Cleveland, Ohio, United States of America; Stanford University School of Medicine, United States of America

## Abstract

The cranial bones and dermis differentiate from mesenchyme beneath the surface ectoderm. Fate selection in cranial mesenchyme requires the canonical Wnt effector molecule β-catenin, but the relative contribution of Wnt ligand sources in this process remains unknown. Here we show Wnt ligands are expressed in cranial surface ectoderm and underlying supraorbital mesenchyme during dermal and osteoblast fate selection. Using conditional genetics, we eliminate secretion of all Wnt ligands from cranial surface ectoderm or undifferentiated mesenchyme, to uncover distinct roles for ectoderm- and mesenchyme-derived Wnts. Ectoderm Wnt ligands induce osteoblast and dermal fibroblast progenitor specification while initiating expression of a subset of mesenchymal Wnts. Mesenchyme Wnt ligands are subsequently essential during differentiation of dermal and osteoblast progenitors. Finally, ectoderm-derived Wnt ligands provide an inductive cue to the cranial mesenchyme for the fate selection of dermal fibroblast and osteoblast lineages. Thus two sources of Wnt ligands perform distinct functions during osteoblast and dermal fibroblast formation.

## Author Summary

Craniofacial abnormalities are relatively common congenital birth defects, and the Wnt signaling pathway and its effectors have key roles in craniofacial development. Wntless/Gpr177 is required for the efficient secretion of all Wnt ligands and maps to a region that contains SNPs strongly associated with reduced bone mass, and heterozygous deletion is associated with facial dysmorphology. Here we test the role of specific sources of secreted Wnt proteins during early stages of craniofacial development and obtained dramatic craniofacial anomalies. We found that the overlying cranial surface ectoderm Wnts generate an instructive cue of Wnt signaling for skull bone and skin cell fate selection and transcription of additional Wnts in the underlying mesenchyme. Once initiated, mesenchymal Wnts may maintain Wnt signal transduction and function in an autocrine manner during differentiation of skull bones and skin. These results highlight how Wnt ligands from two specific tissue sources are integrated for normal craniofacial patterning and can contribute to complex craniofacial abnormalities.

## Introduction

The bones of the skull vault develop in close contact with the embryonic skin to enclose the brain. In the mouse embryo, both bone-forming osteoblasts and skin-forming dermal fibroblasts are derived from cranial neural crest and paraxial mesoderm [1]. At E11.5, cranial dermal fibroblast progenitors undergo specification beneath the surface ectoderm while osteoblast progenitors are specified in a deeper layer of cranial mesenchyme above the eye [2]–[4]. Subsequently, osteoblast progenitors proliferate and migrate apically beneath the dermal progenitors [1], [4]. Both cell types secrete collagen as extracellular matrix, but skull bones provide physical protection for the brain, while the overlying dermis lends integrity to the skin and houses the epidermal appendages [5].

Both paracrine and autocrine intercellular signals function in early bone and skin development. In craniofacial bone formation the mesenchyme sets the timing of ossification [6], [7], while the surface ectoderm functions in a permissive manner [8]. Likewise, during skin formation ectodermal signals are essential for formation of the trunk hair-follicle forming dermis [9], [10], but the cranial dermal mesenchyme determines epidermal appendage identity such as hair or feather [11]. Further delineation of specific ectoderm-mesenchyme signaling during early development of the bone and dermis is required to overcome challenges in the engineering of replacement connective tissues.

Mesenchymal canonical Wnt/β-catenin signal transduction is essential in the specification and morphogenesis of both craniofacial dermis and bone [2], [3], [12]–[15], and dysregulation in components of such signaling pathways is associated with diseases of bone and skin [1], [2], [16]–[18]. Wntless (*Wls*) functions specifically in trafficking of Wnt ligands and is required for the efficient secretion of Wnt ligands. [2]–[4], [19]–[28]. Genetic deletion of *Wls* in mice is likely to dramatically reduce the levels of active Wnt ligands and can recapitulate phenotypes obtained by genetic ablation of Wnt ligands in mice [1], [4], [29]. Wnt ligand binding to target cell surface receptors (Fzd and LRP5/6) results in nuclear translocation of β-catenin, which binds to TCF/LEF transcription factors and activates expression of downstream targets. Certain Wnt ligands also activate the non-canonical Wnt/Planar Cell Polarity (PCP) pathway, which influences cellular movements [5], [30], [31]. β-catenin is essential in osteoblast differentiation and inhibition of chondrogenesis [6], [7], [12]–[14]; however, deletion of individual Wnt ligands resulted only in mild effects on bone differentiation [8], [32], [33]. β-catenin is also a central regulator of early dermal specification [3], [9], [10], [34], [35], and roles for Wnt ligands so far have only been directly shown later during hair follicle initiation [9], [11], [36]. In bone and skin development, redundant functions of multiple Wnts may compensate for deletion of individual ligands. Conventional knockouts of individual ligands removed Wnt expression from all cells in the embryo, and have confounded the identification of tissue sources of Wnt ligands in bone and skin development. Thus, the relative contributions from different sources of Wnt ligands for fate selection in cranial mesenchyme remain unknown.

Previous limitations were the lack of genetic tools to spatiotemporally manipulate early surface ectoderm and mesenchyme, and an inability to circumvent the intrinsic redundancy of Wnt ligands. We took a conditional approach to ablate the efficient secretion of Wnt ligands from either surface ectoderm or cranial mesenchyme prior to fate selection of the cranial bone and dermal lineages. Our findings provide key insights into how local developmental signals are utilized during morphogenesis to generate the cranial bone and dermal lineages.

## Results

**Figure 1:**
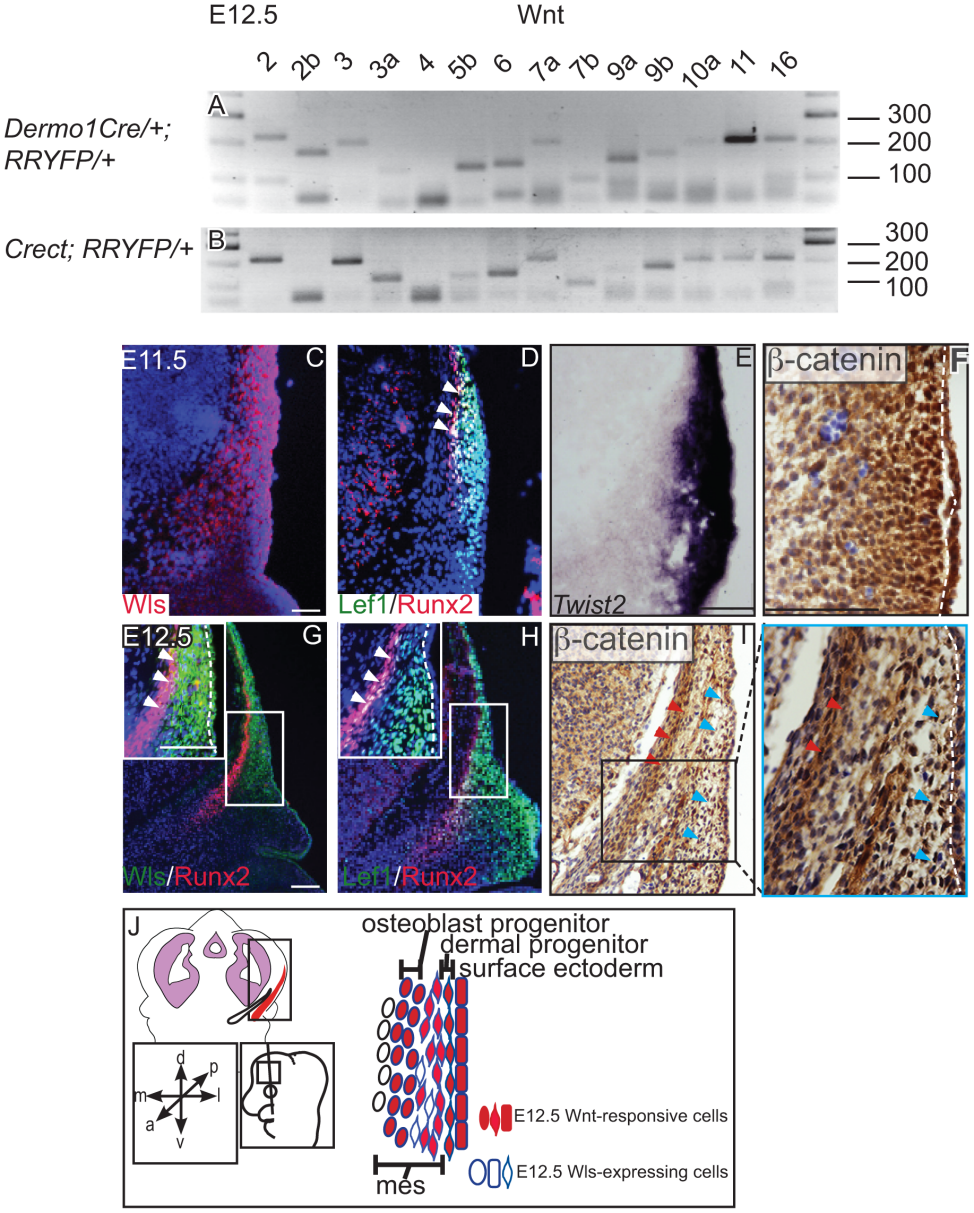
Expression of Wnt ligands, Wntless, and Wnt signaling response in cranial ectoderm and mesenchyme. (A, B) RT-PCR for individual Wnt ligands was performed on cDNA from purified mouse embryonic cranial mesenchyme and surface ectoderm. (C, D G, H) Indirect immunofluorescence with DAPI counterstained nuclei (blue), (E) *in situ* hybridization, or immunohistochemistry (F, I) was performed on coronal mouse embryonic head sections. (G, H, I) Boxes indicate region in insets at higher magnification. White arrowheads indicate co-expression of (G) Wls/Runx2 or (D,H) Lef1/Runx2, (I) red arrowheads indicate osteoblast progenitors, and blue arrowheads indicate dermal progenitors. (F–I) White hatched lines demarcate ectoderm from mesenchyme. (J) Summary scheme of E12.5 supraorbital cranial mesenchyme. (J) Embryonic axes, figure depicts lateral view of embryonic head, region of interest in sections used in figures are shown. Scale bars represent 100 µm.

We found that the genes for most Wnt ligands were expressed in the cranial mesenchyme (Figure 1A) and surface ectoderm (Figure 1B) during the specification of two separate lineages such as cranial osteoblast and dermal fibroblasts in E12.5 mouse embryos (Figure S1, S7, Table 1). To identify the cells with the potential to secrete Wnt ligands, we examined the spatiotemporal expression of Wls, the Wnt ligand trafficking regulator. We detected Wls protein expression from E11.5-E12.5 in the cranial surface ectoderm and in the underlying mesenchyme (Figure 1C, G). Both the Runx2-expressing cranial bone progenitor domain and the *Dermo1/Twist2*-expressing dermal progenitor domain expressed Wls [3], [37] (Figure 1C, D, E, G). Wnt signaling activation was also visualized in the cranial ectoderm, bone and dermal progenitors by expression of target gene, Lef1 and nuclear localized β-catenin (Figure 1D, F, H, I). During specification of cranial bone and dermis, ectodermal and mesenchymal tissues secreted Wnt ligands, and the dermal and bone progenitors actively transduced Wnt signaling via β-catenin (Figure 1J).

**Table 1:** Primer sequences for RT-PCR of mouse Wnt genes.

Ligand	Primers	Tm (1 um Primer)	Size	Intron-exon junction	GENEBANK	Coordinates - Dec. 2011 (GRCm38/mm10)
Wnt1 F	ATGAACCTTCACAACAACGAG	59	205	yes	145386529	chr15:98,791,925–98,791,945
Wnt1 R	GGTTGCTGCCTCGGTTG	63				chr15:98,792,568–98,792,584
Wnt2 F	CTGGCTCTGGCTCCCTCTG	64	221	yes	242397431	chr6:18,027,993–18,028,013
Wnt2 R	GGAACTGGTGTTGGCACTCTG	64				chr6:18,030,246–18,030,265
Wnt2b F	CGTTCGTCTATGCTATCTCGTCAG	63	170	no	118130343	chr3:104,953,154–104,953,177
Wnt2b R	ACACCGTAATGGATGTTGTCACTAC	62				chr3:104,953,009–104,953,033
Wnt3 F	CAAGCACAACAATGAAGCAGGC	65	200	yes	254028153	chr11:103,811,566–103,811,587
Wnt3 R	TCGGGACTCACGGTGTTTCTC	66				chr11:103,812,431–103,812,451
Wnt3a F	CACCACCGTCAGCAACAGCC	68	119	yes	226958415	chr11:59,275,170–59,275,189
Wnt3a R	AGGAGCGTGTCACTGCGAAAG	65				chr11:59,256,477–59,256,497
Wnt4 F	GAGAAGTGTGGCTGTGACCGG	67	80	yes	342672048	chr4:137,295,527–137,295,547
Wnt4 R	ATGTTGTCCGAGCATCCTGACC	66				chr4:137,295,669–137,295,690
Wnt5a F	CTCCTTCGCCCAGGTTGTTATAG	64	97	yes	371940977	chr14:28,511,918–28,511,940
Wnt5a R	TGTCTTCGCACCTTCTCCAATG	66			371940978	chr14:28,513,276–28,513,297
Wnt5b F	ATGCCCGAGAGCGTGAGAAG	64	128	yes	158321898	chr6:119,440,354–119,440,373
Wnt5b R	ACATTTGCAGGCGACATCAGC	64			415702095	chr6:119,433,821–119,433,841
Wnt6 F	TGCCCGAGGCGCAAGACTG	72	130	yes	119672921	chr1:74,782,215–74,782,234
Wnt6 R	ATTGCAAACACGAAAGCTGTCTCTC	66				chr1:74,782,566–74,782,590
Wnt7a F	CTTCATGTTCTCCTCCAGGATCTTC	64	205	yes	144227223	chr6:91,366,308–91,366,332
Wnt7a R	CGACTGTGGCTGCGACAAG	64				chr6:91,394,575–91,394,593
Wnt 7b F	TCTCTGCTTTGGCGTCCTCTAC	64	96	yes	254692921	chr15:85,581,391–85,581,412
Wnt 7b R	GCCAGGCCAGGAATCTTGTTG	63			254692925	chr15:85,559,067–85,559,087
Wnt 8a F	ACGGTGGAATTGTCCTGAGCATG	66	106	yes	165972302	chr18:34,542,924–34,542,946
Wnt 8a R	GATGGCAGCAGAGCGGATGG	68				chr18:34,544,809–34,544,828
Wnt 8b F	TTGGGACCGTTGGAATTGCC	68	173	yes	225637541	chr19:44,509,616–44,509,635
Wnt 8b R	AGTCATCACAGCCACAGTTGTC	61				chr19:44,510,510–44,510,531
Wnt 9a F	GCAGCAAGTTTGTCAAGGAGTTCC	66	137	yes	70778750	chr11:59,328,673–59,328,696
Wnt 9a R	GCAGGAGCCAGACACACCATG	67				chr11:59,330,929–59,330,949
Wnt 9b F	AAGTACAGCACCAAGTTCCTCAGC	64	166	yes	238231387	chr11:103,732,048–103,732,071
Wnt 9b R	GAACAGCACAGGAGCCTGACAC	65				chr11:103,731,160–103,731,181
Wnt 10a F	CCTGTTCTTCCTACTGCTGCTGG	65	200	yes	229094723	chr1:74,792,245–74,792,267
Wnt 10a R	CGATCTGGATGCCCTGGATAGC	68				chr1:74,793,502–74,793,523
Wnt 10b F	TTCTCTCGGGATTTCTTGGATTC	63	115	yes	274317542	chr15:98,774,188–98,774,210
Wnt 10b R	TGCACTTCCGCTTCAGGTTTTC	66				chr15:98,772,904–98,772,925
Wnt 11 F	CTGAATCAGACGCAACACTGTAAAC	63		no		chr7:98,846,405–98,846,429
Wnt 11 R	CTCTCTCCAGGTCAAGCAGGTAG	63	205		254750613	chr7:98,846,587–98,846,609
Wnt 16 F	AGTAGCGGCACCAAGGAGAC	63	225	yes	255683340	chr6:22,289,022–22,289,029; chr6:22,240,918–22,240,939
Wnt 16 R	GAAACTTTCTGCTGAACCACATGC	65				chr6:22,291,095–22,291,118

**Figure 2:**
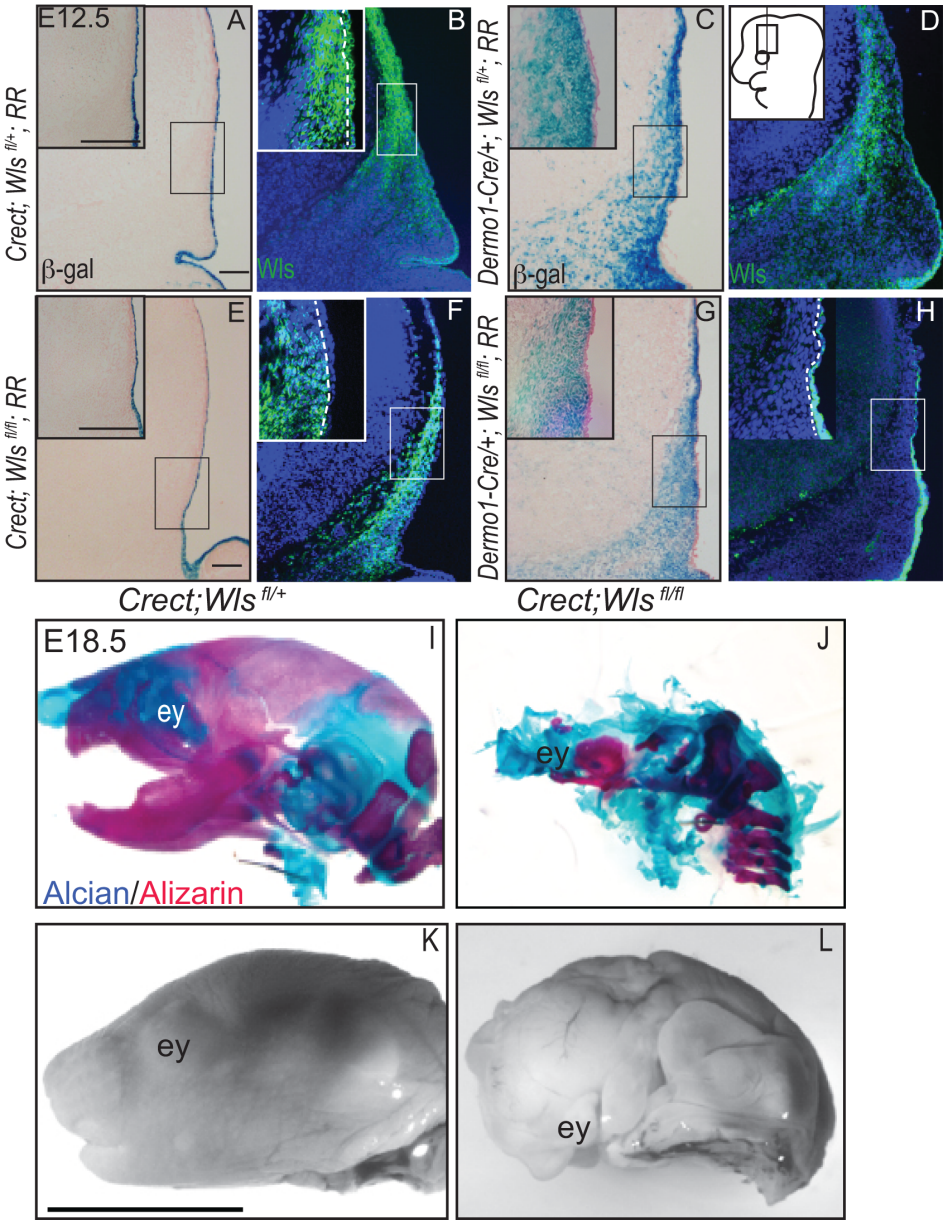
Deletion of *Wntless* in the cranial ectoderm and mesenchyme. (A, C, E, G) β-galactosidase staining with eosin counterstain or (B, D, F, H) indirect immunofluorescence with DAPI-stained nuclei (blue) was performed on coronal mouse E12.5 head sections. (A–H) Box outlines indicate region in inset and white hatched line in insets demarcates cranial ectoderm from mesenchyme. (I–L) Lateral view of whole-mount skeletal preps or gray-scaled bright field images of embryonic mouse heads. Ey, eye. Scale bars for sections represent 100 µm. Scale bar (K) for whole mount pictures (I–L) represents 5 mm. Diagram inset in (D) depicts lateral view of embryonic head with box outlining region of interest.

To dissect the requirements of ectodermal and mesenchymal Wnt signals, we generated mutant mice with conditional deletion of *Wls* [38] in the early surface ectoderm using *Crect* [39] and in the whole cranial mesenchyme using *Dermo1Cre* [40]. *Crect* efficiently recombined the Rosa26 LacZ Reporter (RR) in the cranial ectoderm by E11.5 (Figure S4K), but left Wls protein expression intact in the mesenchyme (Figure 2A, E, B, F) [41]. *Dermo1Cre* recombination showed β-galactosidase activity and *Wls* deletion restricted to the cranial mesenchyme and meningeal progenitors at E12.5, and Wls protein was still expressed in the ectoderm in mutants (Figure 2C, D, G, H).

First, we compared the extent to which *Wls* deletion from ectoderm or mesenchyme affected formation of the craniofacial skeleton. E18.5 *Crect; RR; Wls ^fl/fl^* mutant embryos, which experienced perinatal lethality, demonstrated a hypoplastic face with no recognizable upper or lower jaw most likely due to decrease in cell survival of branchial arch mesenchyme (Figure S5). In the remaining tissue, facial mesenchyme patterning was grossly comparable to controls for most of the markers examined (Figure S5). Notably, the mutants showed no sign of mineralization in the skull vault (Figure 2I–L). The later deletion of *Wls* from the ectoderm using the *Keratin14Cre* line resulted in comparable skull bone ossification as controls (Figure S2). *Dermo1Cre*; *RR; Wls ^fl/fl^* mutant embryos exhibited lethality after E15.5, which precluded assessment of skeletogenesis by whole-mount. We generated *En1Cre/+; RR; Wls ^fl/fl^* mutants, using a Cre that recombines in early cranial mesenchyme but lacks activity in meningeal progenitors (Figure S3 E′, F′) [3]. *En1Cre/+; RR; Wls ^fl/fl^* mutants survived until birth, and demonstrated reduced bone differentiation and mineralization (Figure S3) as well as intact dermis in the supraorbital region with hair follicles (Figure S3). The more severe arrest in *Crect; RR; Wls ^fl/fl^* mutants (Figure 2) suggested ectoderm *Wls* appears to play an earlier role than mesenchymal Wls in cranial development.

**Figure 3:**
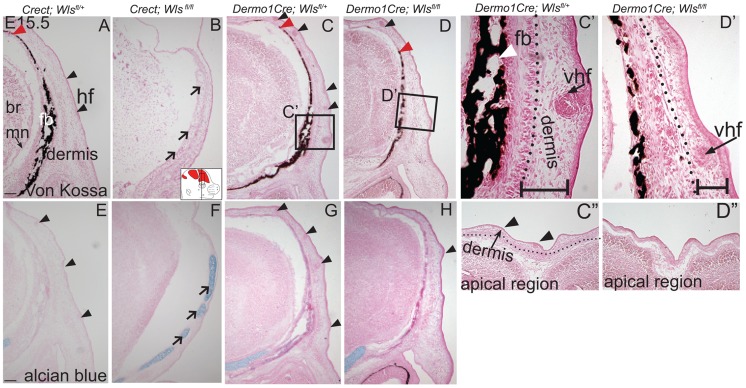
Distinct requirements for *Wntless* in the cranial ectoderm and mesenchyme. (A, B, C, D, C′, D′) Von Kossa staining, or (E–H) alcian blue staining was performed on coronal mouse embryonic head sections and counterstained with eosin. Br, brain, fb, frontal bone, vhf, supraorbital vibrissae hair follicle, mn, meningeal progenitors. Black arrowheads indicate guard hair follicles (hf), red arrowheads indicate dorsal extent of ossified frontal bone, and open black arrows indicate ectopic cartilage. (C′, D′ C″, D″) Black dotted line demarcates the lower limit of the dermal layer and the black bracket shows dermal thickness. Diagrams inset (B) figure depicts lateral view of E15.5 embryonic head with plane of section and region of interest. Red regions in diagram represent bone primordia. Scale bars (A,E) represent 100 µm.

We next examined the effects of ectoderm or mesenchyme *Wls* deletion on cranial bone and dermal development by histology. We found Von Kossa staining for bone mineral was absent in *Crect; RR; Wls ^fl/fl^* mutants (Figure 3A, B). The thin domain of mesenchyme above the eye in mutants appeared undifferentiated and showed no condensing dermal cells or early stage hair follicles. Additionally, the baso-apical expansion of both dermis and bone was evident by E15.5 in controls, but not in the thin cranial mesenchyme of mutants (Figure 3A–B red arrowhead). Although ossification was absent, we observed the presence of thin nodules of ectopic, alcian blue-stained cartilage (Figure 3E–F). Therefore the result of *Wls* deletion in the ectoderm was an absence of skull ossification and hair-inducing dermis, a failure of baso-apical expansion of mesenchyme, and the presence of ectopic chondrocyte differentiation. By comparison, *Dermo1Cre*; *RR; Wls ^fl/fl^* mutants showed a reduction in mineralized bone (Figure 3C–D) without ectopic cartilage formation (Figure 3 G–H). The mutant mesenchyme nonetheless condensed and formed sufficient hair-follicle generating dermis in the supraorbital region to support the supraorbital vibrissae hair follicle and fewer primary guard hair follicles (Figure 3 C, D, C′, D′, black arrowheads). Compared to the control apical region of the head, the mutant lacked sufficient condensed dermal layer to support normal number and differentiation of hair follicles (Fig. 3 C″, D″). Reduced mineralization without ectopic chondrogenesis as well as hair-follicle formation were also present in *En1Cre/+*; *Wls ^fl/fl^* mutants (Figure S3). Our data suggest that *Wls* deletion using the *Dermo1Cre* resulted in diminished bone mineralization with thinner dermis and fewer hair follicles.

**Figure 4:**
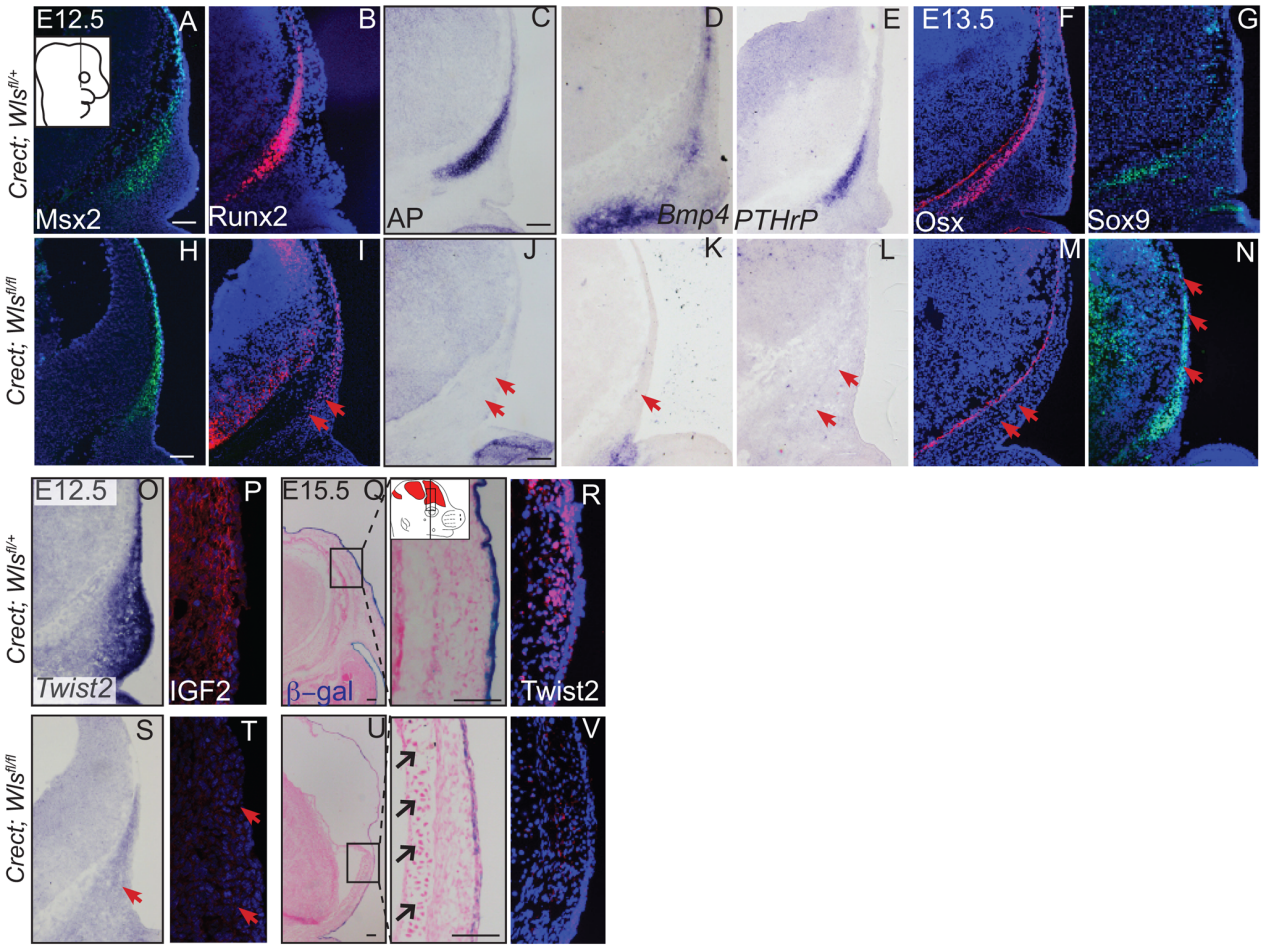
Ectoderm deletion of *Wntless* leads to loss of cranial bone and dermal lineage markers in the mesenchyme. Indirect immunofluorescence with DAPI-stained (blue) nuclei was performed on coronal mouse embryonic head sections at E12.5 or as indicated (A,B, F, G, H, I, M, N, P, R, T, V). Alkaline Phosphatase staining (C, J), in situ hybridization (D, E, K, L, O, S), or β-galactosidase staining with eosin counterstain (Q, U) was performed on coronal tissue sections. Diagram in (A) demonstrates plane of section and region of interest for E12.5-E13.5 (A–T). Box and dashed lines in (Q, U) demonstrate the region of high magnification, and β-galactosidase stained sections were included for perspective for (R, V). Diagram inset in high magnification photograph from (Q) shows plane of section and region of interest for E15.5. Red arrows indicate changes in marker expression and black arrows in (U) high magnification indicate ectopic cartilage. Scale bars represent 100 µm.

Deletion of *Wls* from the ectoderm resulted in complete absence of skull vault mineralization with failure of dermis formation, pointing to early defects in formation of the two lineages. Therefore we tested if cranial mesenchyme undergoes proper patterning, fate selection, and differentiation in the absence of *Wls*. Msx2 and Dlx5 that are early markers of skeletogenic patterning in cranial mesenchyme were expressed in *Crect; Wls ^fl/fl^* mutants (Figures 4A, H, S4). The number of Msx2^+^ progenitor cells was not significantly different in controls and mutants (191±9.4 in controls and 206±24 in mutants, P-value = 0.23). However, few Runx2^+^ osteoblast progenitors formed in *Crect; RR; Wls ^fl/fl^* mutant embryos, and expression shifted directly beneath the surface ectoderm (Figure 4B, I). During subsequent differentiation, condensing osteoblast progenitors express alkaline phosphatase (AP; Figure 4C, S4), but ectoderm Wnt-secretion deficient embryos lacked AP activity entirely (Figure 4J, S4). Markers of early osteoblast progenitors from other signaling pathways, *Bmp4* and *PTHrP* (Figure 4D–E, K–L) were also absent in mutants, suggesting an arrest in osteoblast progenitor differentiation. The block was persistent as committed osteoblast progenitors expressing Osx were present in controls but not mutants (Figure 4F, M). Cell survival was not affected in the cranial mesenchyme prior to changes in marker expression (Figure S4A–D). We did not find significant difference in cell proliferation of the underlying mesenchyme (47%±4 in controls and 51%±2; P-value = 0.12). Whereas chondrocytes expressed Sox9 only at the skull base in controls, in mutants, ectopic Sox9-expressing chondrocyte progenitors and cartilage formed within the frontal bone domain (Figure 4G, N, Q, U). In spite of the effect of ectoderm-*Wls* deletion on mesenchyme, surface ectoderm expression of the differentiation marker, Keratin 14 (K14) was unaffected (Figure S4E,F). Next, we examined formation of dermal fibroblast progenitors in *Crect; RR; Wls ^fl/fl^* mutant embryos. Cranial dermal fibroblast progenitors expressed the markers, *Twist2* [3], [37] and Insulin Growth factor 2 (IGF2) by E12.5 in supraorbital mesenchyme (Figure 4O, P), but mutant embryos lacked *Twist2* and IGF2 expression (Figure 4S, T). Twist2 expression became more progressively restricted to upper dermal fibroblasts during differentiation in controls, but was completely absent from cranial supraorbital mesenchyme of mutants (Figure 4 R, V). The altered cell fate marker expression at E12.5 (Figure 4, S4 I, J) immediately after deletion of ectoderm *Wls* (Figure S4K) was indicative of primary defects in mesenchymal cell fate selection. Together, our data suggest ectoderm Wnts form a non-cell autonomous inductive signal to the underlying mesenchyme for specification of osteoblast and dermal fibroblast progenitors, and for repression of chondrogenesis.

**Figure 5:**
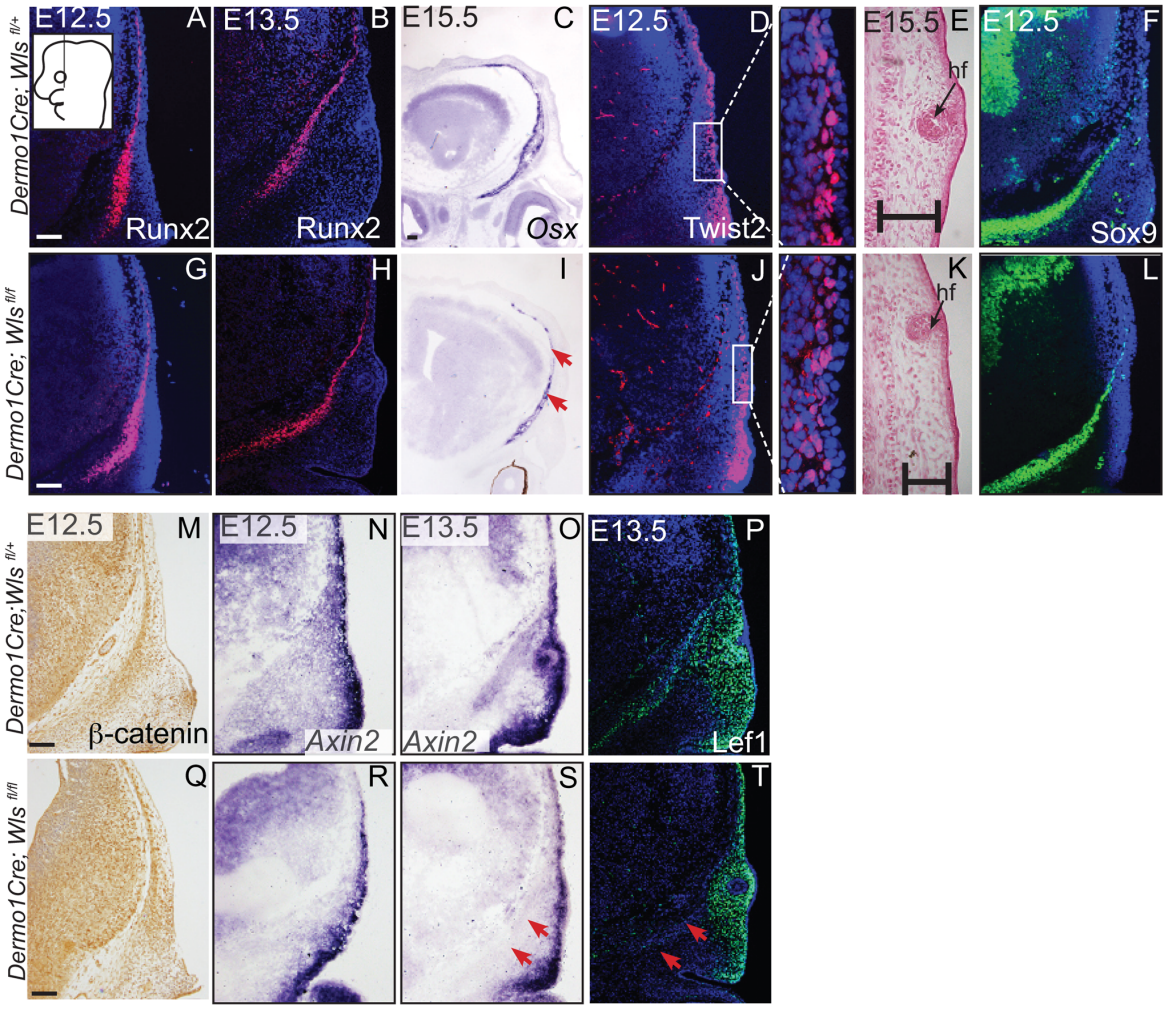
Mesenchyme deletion of *Wntless* leads to diminished differentiation and Wnt responsiveness in the bone lineage. Indirect immunofluorescence with DAPI-stained (blue) nuclei (A, B, D, F, G, H, J, L, P, T) and immunohistochemistry (M,Q) was performed on coronal mouse embryonic head sections In situ hybridization (C, I, N, O, R, S) or eosin counterstain (E, K), was performed on coronal tissue sections of embryonic murine heads at the indicated stages. Diagram in (A) demonstrates plane of section and region of interest for E11.5-E12.5. Box in (D, J) demonstrate the region of high magnification. (I, S, T) Red arrows highlight changes in marker expression in osteoprogenitor domain. (E,K) vhf: subraorbital vibrissae hair follicle and black bracket indicates the dermal layer. (A,G) Scale bars represent 100 µm.

Next, we determined if mesenchyme *Wls* deletion resulted in a later defect in differentiation of cranial bone and dermal fibroblast progenitors. In *En1Cre*; *RR; Wls ^fl/fl^* mutants, Runx2 expression in osteoblast progenitors was intact without ectopic Sox9 expression, but showed diminished expression of the skeletal differentiation marker, *Osx* and ossification (Figure S3). Wnt responsiveness by *Axin2* expression was comparable in control and mutant cranial mesenchyme at E14.5 (Figure S3). In *Dermo1Cre*; *RR; Wls ^fl/fl^* mutants, Runx2 expression was also unaffected during fate selection stages (Figure 5A, G, B, H). However, during later osteoblast progenitor differentiation (E15.5), *Osx* was diminished in mutants at E15.5 (Figure 5C, I). In dermal progenitors undergoing specification, *Twist2* expression was unaffected (Figure 5D,J), and surface ectoderm differentiation marker, K14, was appropriately expressed (Figure S6C, D). Additionally at later stages in the mutant, we observed thinner dermis, which was sufficient to support initiation of fewer guard hair follicles (data not shown) and supraorbital vibrissae hair follicle formation (Figs. 3C, D; 5E, K). Furthermore, no ectopic expression of Sox9 occurred in mesenchyme *Wls*-deficient mutants (Figs. 5F, L). Deletion of mesenchyme-*Wls* did not lead to decrease in cell survival as monitored by expression of activated-Caspase3 (Figure S6A–B). Prior to E15.5, cell proliferation of osteoblast, dermal, and surface ectoderm progenitors was not significantly different from controls (Figure S6). Based on *Dermo1Cre-* and *En1Cre-* deletion of *Wls*, mesenchyme-derived Wnt ligands are not required for differentiation of dermal progenitors but are indispensable for later differentiation of osteoblast progenitors. Next, we tested the spatiotemporal requirement for mesenchyme *Wls* in Wnt signaling transduction. Nuclear β-catenin and *Axin2* expression were comparable in the mesenchyme of mutants during fate selection stages at E12.5 (Figure 5M, N, Q, R). As differentiation occurs, expression of *Axin2* and Lef1 was selectively diminished in the osteoblast progenitor domain of mesenchyme-*Wls* mutants compared to the controls (Figure 5O, P, S, T). Thus, mesenchyme Wnt ligands appeared to be important in mesenchyme Wnt signal transduction during osteoblast differentiation and ossification as opposed to earlier lineage specification events.

**Figure 6:**
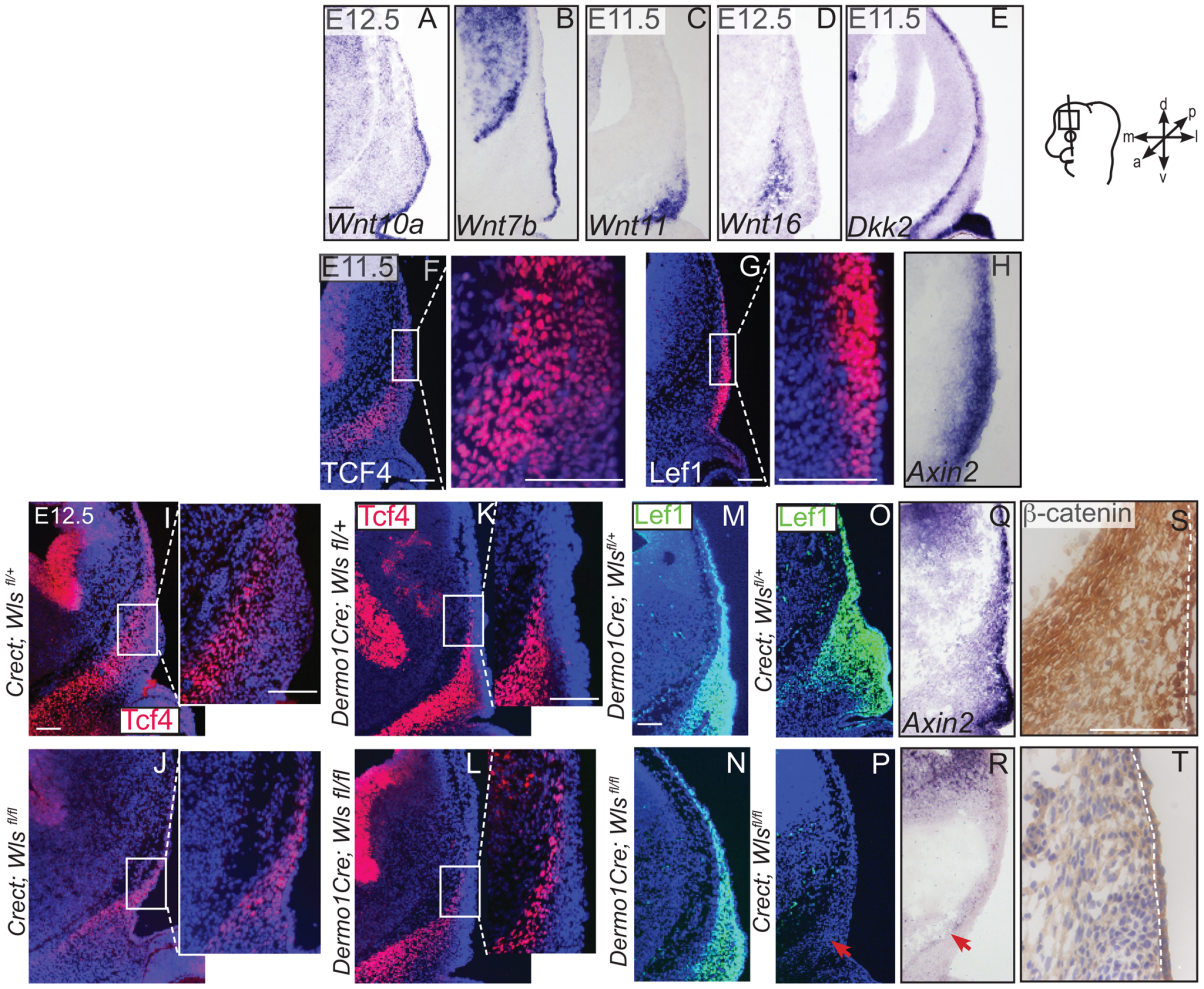
Generation of Wnt responsiveness in the cranial mesenchyme. In situ hybridization (A–E, H, Q, R), immunohistochemistry (S, T) or indirect immunofluorescence with DAPI-stained nuclei (blue) was performed on coronal mouse embryonic head sections (F, G, I–P). (S, T) White dotted line demarcates ectoderm from mesenchyme. Embryonic head diagram depicts region of interest and plane of section. Embryonic axes for the sections are presented. Scale bars represent 100 µm.

Next, we examined the source of Wnts for the onset of Wnt responsiveness in the mesenchyme. During dermal and osteoblast progenitor cell fate selection, Wnt ligands, inhibitors, and target genes are expressed in spatially segregated patterns. *Wnt10a* and *Wnt7b* were expressed in surface ectoderm (Figure 6A–B), *Wnt11* was expressed in sub-ectodermal mesenchyme (Figure 6C), and *Wnt16* mRNA was expressed in medial mesenchyme (Figure 6D). Notably, the soluble Wnt inhibitor, *Dickkopf2* (*Dkk2*) mRNA was localized to the deepest mesenchyme overlapping with cranial bone progenitors (Figure 6E). Wnt ligands can induce nuclear translocation of β-catenin in a dose-dependent manner leading to the expression of early target genes [42], [43]. At E11.5, expression of nuclear β-catenin was present in both dermal and osteoblast progenitors, and the highest intensity of nuclear localization was found in the surface ectoderm and dermal mesenchyme (Figure 1F). Wnt target genes Lef1, Axin2, and TCF4 were patterned in partially complementary domains. Expression of Tcf4 protein was visible in the skeletogenic mesenchyme (Figure 6F). Tcf4 expression expanded into the mesenchyme under the ectoderm in ectoderm *Wls*-deficient mutants (Figure 6I–J) and was diminished in mesenchyme *Wls*-deficient mutants compared to controls (Figure 6K–L). Lef1 and Axin2 were expressed at the highest intensity in the dermal progenitors beneath the ectoderm (Figure 6 G, H). At E12.5, Lef1 expression was completely abolished in the mesenchyme of ectoderm-*Wls* mutants, but was comparable to controls in the absence of mesenchyme-*Wls* (Figure 6M–P). The onset of Wnt signaling response in the mesenchyme as measured by Lef1, Axin2, and nuclear β-catenin expression (Figure 6O–T) required ectoderm *Wls*. By contrast, no single tissue source of Wnt ligands was required to maintain TCF4 expression.

**Figure 7:**
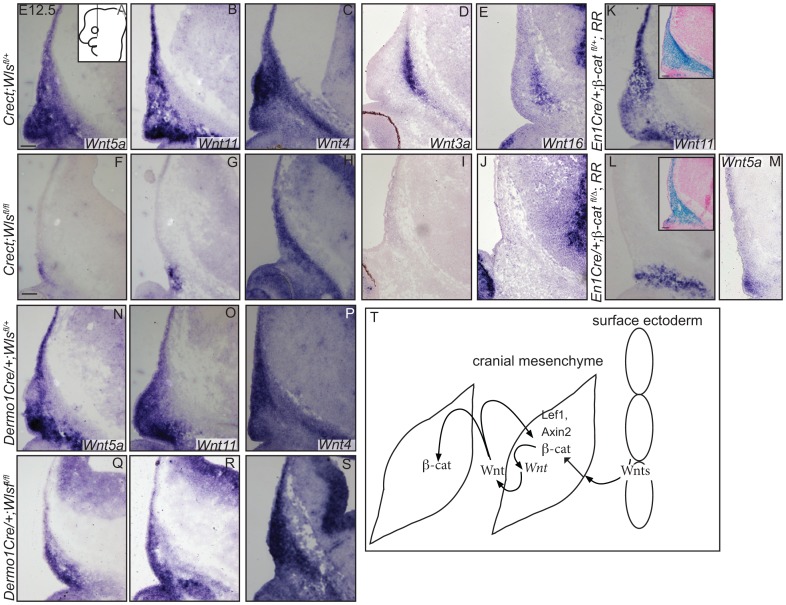
Mesenchyme Wnt ligand expression is dependent on ectoderm *Wls* and mesenchymal *β-catenin*. (A–S) *In situ* hybridization was performed on coronal mouse embryonic head sections. Diagram of embryonic head in (A) inset depicts region of interest and plane of section. Insets in (K, L) show β-galactosidase staining and eosin counterstaining on serial sections. (T) A working model for role of tissue sources of Wnt ligands during cranial mesenchymal lineage fate selection. Scale bars represent 100 µm.

Finally, we tested whether cranial surface ectoderm Wnt ligands regulate the onset of Wnt ligand mRNA expression in the underlying mesenchyme (Figure 7). The non-canonical ligands Wnt5a and Wnt11 were expressed in cranial mesenchyme, with the highest expression corresponding to dermal progenitors. Wnt4, which signals in canonical or non-canonical pathways [44], was expressed strongly in dermal progenitors, as well as in osteoblast progenitors and in the skull base (Figure 7A–C). Wnt3a and 16, which signal in the canonical pathway via β-catenin and have roles in intramembranous bone formation, were expressed medially in the cranial mesenchyme containing cranial bone progenitors (Figure 7D, E) [12]–[14], [45]. Expression of *Wnt5a Wnt11, Wnt3a, Wnt16* mRNAs was absent from the mesenchyme of *Crect; RR; Wls ^fl/fl^* mutants whereas some *Wnt4* expression was maintained (Fig. 7F–J). *En1Cre* deletion of β-catenin in the cranial mesenchyme [12] also resulted in an absence of *Wnt5a and Wnt11* expression, except in a small portion of supraorbital lineage-labeled mesenchyme, suggesting a phenocopy of *Crect;Wls* mutants (Figure 7K, L, M). In contrast, *Wnt5a, Wnt11, and Wnt4* expression were present in the *Dermo1Cre; RR; Wls^fl/fl^* mutants (Figure 7N–S). However, the Wnt-expressing domains were smaller and only located close to the surface ectoderm, but nonetheless were lineage-labeled (Figure 7E–G, L–N; not shown). Thus, consistent with a role as initiating factors, ectoderm Wnt ligands and mesenchyme β-catenin were required for expression of certain Wnt ligands in the cranial mesenchyme during lineage selection. Mesenchymal Wnt ligands may in turn be required later for osteoblast differentiation (Figure 7T).

## Discussion

Here we obtained data suggesting that ectodermal and mesenchymal Wnts function distinctly in early dermal and osteoblast progenitor specification and differentiation. Wnt ligands are expressed in the cranial surface ectoderm and mesenchyme, and ectoderm Wnts are required to generate an inductive cue for the specification of multiple lineages in the cranial mesenchyme. The dermal progenitors and osteoblast progenitors closest to the ectoderm experience the highest concentrations of nuclear β-catenin, in response to Wnt ligands from overlying ectoderm. Subsequent differentiation of osteoblast and dermal fibroblast progenitors requires *Wls* from the mesenchyme. Thus our study demonstrates that two different sources of Wnt signals coordinate to form two separate lineages, bone and dermis.

We present evidence to demonstrate that ectoderm Wnts generate an inductive cue of Wnt signaling in the mesenchyme to specify cranial bone and dermal lineages. The mechanism remains elusive; however, there are at least three possible models. First, the spatial segregation of Wnt pathway transcription cofactors such as Lef1 and TCF4, partially by lineage, provides a mechanism to generate different lineage programs. Second, a threshold-dependent model may also exist to generate multiple lineages from the same signal. At E11.5–12.5, dermal progenitors are closest to the ectoderm Wnt source and exhibit the highest Wnt signaling reporter activity and markers induced by constitutive activation of β-catenin in mesenchyme (Figure 1) [3], [9], [46]. High levels of Wnt pathway activity preclude osteoblast marker expression in the mesenchyme [12]. Consistently, osteoblast progenitors are present farther away from the ectoderm in an overlapping domain to at least one Wnt inhibitor, *Dkk2* [47] (Figure 6E). Finally, the osteoblast response to ectodermal Wnts may be indirect; osteoblast progenitors may require a separate signal relayed from dermal progenitors. Future genetic experiments with new reagents will be required to distinguish between these models and test direct or indirect requirements of Wnt sources in osteoblast and dermis formation.

During fate selection of cranial dermal and osteoblast progenitors, upstream ectodermal Wnt ligands initiate expression of a subset of mesenchymal Wnt ligands via β-catenin. Ectoderm Wnts also act upstream of mesenchyme Wnts in mouse limb development [48]. Here, ectoderm Wnts act in a temporally earlier role than mesenchyme Wnts, and other studies support a direct relationship. In at least one instance, mesenchyme Wnt ligands are direct targets of canonical Wnt signaling [49]. Alternatively, ectoderm and mesenchyme Wnts may signal in parallel pathways to the mesenchyme. The signal that acts upstream to initiate Wnt ligand expression in the cranial ectoderm remains unknown.

We report here that osteoblast differentiation requires distinct Wnt signals from surface ectoderm and mesenchyme. β-catenin deletion in the ectoderm did not inhibit skull bone mineralization [39], so autocrine effects of *Wls* deletion on the ectoderm were unlikely to contribute to the skull phenotype. However, removal of surface ectoderm *Wls* resulted in ectopic chondrogenesis (Figure 3), which phenocopied mesenchymal *β-catenin* deletion [12]. In contrast, mesenchymal *Wls* deletion did not result in ectopic cartilage formation, suggesting repression of chondrogenesis in cranial mesenchyme requires an early, ectoderm Wnt signal. Our results thus implicate β-catenin here as a Wnt pathway factor that acts in the nucleus to repress chondrogenesis and functions downstream of ectoderm ligands. Ectoderm Wnt ligands thus provide an inductive cue acting on osteoblast progenitors while the cells are closest to the ectoderm. Indeed, later deletion of *Wls* from the ectoderm using the K14Cre line did not give rise to a skull bone ossification phenotype (Figure S2). During osteoblast progenitor differentiation, *Wls* deletion with *Dermo1Cre* resulted in a similar but more severe differentiation arrest than the more restricted *En1Cre*. Consistently, using a different *Wls* mutant allele, deletion of mesenchymal Wnts led to absence of osteoblast differentiation expression and reduced cell proliferation [50]. We show that the mesenchyme Wnts maintain the differentiation process but require an inductive ectoderm Wnt signal.

We demonstrate that dermal progenitors require ectodermal *Wls* for specification and mesenchymal *Wls* for normal differentiation (Figs. 4–5). Cranial dermal progenitors located beneath the ectoderm require β-catenin for specification [3], but the tissue contribution of Wnt sources remained previously undetermined. Here, a mesenchymal Wls source is indispensable in the dermal lineage for normal differentiation, thickness, and hair follicle patterning. Previous reports in murine trunk skin development suggested that ectoderm Wnts alone are essential in hair follicle induction [9], [10]. Differential requirements may exist for mesoderm-derived trunk dermal progenitors and cranial neural crest-derived dermal progenitors. Future studies will be needed to uncover the requirements for a mesenchymal Wnt signal in dermal fibroblast differentiation in different parts of the embryo.

Conditional *Wls* deletion resulted in a failure of cranial dermal and osteoblast progenitors to undergo baso-apical extension (Figure 3), a process that occurs independently of β-catenin [12]. Since *Wls* deletion blocked secretion of canonical and non-canonical Wnt ligands, extension defects in the mesenchyme are consistent with known roles for non-canonical Wnt ligands in orienting cell movements [51]. Homozygous null mutants of core planar cell polarity (PCP) components lacked proper skull tissue development and neural tube closure [52]. However, mutants for individual non-canonical Wnt ligands lack a cranial PCP phenotype. In the cranial mesenchyme, non-canonical Wnt5a or Wnt11 ligands were expressed in overlapping expression domains, suggesting the ligands function redundantly [53] (Figure 7). Therefore, the role of PCP signaling remains to be rigorously tested in conditional mutant mice. The non-canonical and canonical Wnt signaling pathways interact extensively. In our study, canonical β-catenin transduction, in response to ectodermal Wnts, initiates non-canonical Wnt ligand expression (Figure 7), consistent with reports from other systems [30], [49], [51]. Our results reinforce the role of non-canonical Wnt ligands in the pathogenesis of craniofacial anomalies [54], [55]. The ability of exogenous non-canonical Wnts to compensate for *Wls* deletion in the baso-apical extension of dermal and osteoblast progenitors remains to be tested.

Our results from tissue-specific deletion of *Wls* have implications in diseases with dysregulation of dermal fibroblasts or osteoblasts, and in understanding the pathogenesis of craniofacial birth defects. Removal of Wls from the ectoderm by E12.5 of mouse development reveals a default state for formation of cartilage in the cranial skeleton and dermis if all Wnt secretion were absent from the ectoderm. This forms an important baseline state that can be used to interpret less severe genetic conditions resulting from loss or mutation of individual Wnt ligands. In this respect, we hypothesize that mutations in the Wnt secretory pathway may underlie diseases of osteoblasts, and dermal fibroblasts, warranting continued investigation into the role of Wnt production in bone and skin formation and homeostasis [15], [17], [18], [45], [56]–[58]. Understanding the signals surrounding osteoblast and dermal fibroblast formation is crucial to meet the demands of engineering appropriate connective tissues.

## Materials and Methods

### Mice and genotyping

Conditional functional studies were conducted using *Crect, Keratin 14Cre; Dermo1Cre, En1Cre, β-catenin deleted, conditional β-catenin floxed* mice [39], [40], [59]–[62]. Mice and embryos were genotyped as described previously. The conditional loss-of-function floxed allele for Wls (Wls^fl/fl^) was described previously [38]. RR/RR mice harboring a *LacZ* transgene downstream of a floxed stop transcription signal in the ubiquitous Rosa26 locus were obtained for lineage tracing [41]. For timed matings the vaginal plug day was assigned as E0.5. At desired time points, embryos were harvested and processed for frozen sections as previously described [34]. For each experiment, at least three to five different mutants with littermate controls from 2–3 litters were analyzed. At least three to five litters were used for all analyses. Case Western Reserve Institutional Animal Care and Use Committee approved all animal procedures.

### In situ hybridization, immunohistochemistry, and histology

Embryos were fixed in 4%PFA, cryopreserved, and sectioned at 8–12 µm. In situ hybridization, β-galactosidase with eosin counter-staining, and immunohistochemistry were performed essentially as described [34], [35]. Alcian blue staining of sections was performed as described. For Von Kossa staining of frozen sections, slides were fixed with 4% PFA, incubated in the dark with 2% silver nitrate, rinsed, exposed to light, and counterstained with eosin. In situ probes for *Twist2* (Eric Olson, Dallas, TX), *Pthrp, Wnt4* (V. Lefebvre, Cleveland, OH), *Wnt5a* (Andrew McMahon, Boston, MA), *Wnt11* (Steve Potter, Cincinnati, OH), *Axin2* (Brian Bai), *BMP4*, *Wnt7b, Dlx5* (Gail Martin, San Francisco, CA), Wnt16 (Yingzi Yang, Bethesda, MD) and *Osx* (Matthew Warmann, Boston, MA) were gifts. For *Wnt10a*, cDNA was amplified from E12.5 RNA using primer F: GCTATTTAGGTGACACTATAGGCGCTCTGGGTAAACTGAAG, primer R: TTGTAATACGACTCACTATAGGGAGAGCCAACCACCTCTCTCA, and in vitro transcription of antisense mRNA with T7 polymerase. For *Dkk2*, PCR primers *DKK2-F(5′* -GACATGAAGGAGACCCATGCCTACG-*3′* and *DKK2-T7R 5′* -TGTAATACGACTCACTATAGGGCATAGATGAGGCACATAACGGAAG-*3′* were used.

Primary antibodies for Runx2, Sox9, Twist2, Lef1, Osx, Msx2, Ki67, IGF2, Wls, and β-catenin (goat anti-Runx2; 1∶20, R&D Biosystems; rabbit anti-Sox9; 1∶100; Millipore; mouse anti-Twist2, 1∶500, Santa Cruz; rabbit anti-Lef1, 1∶100, Abcam; rabbit anti-Osx, 1∶400, Abcam; mouse Msx1/2, 1∶50, DSHB; activated Caspase3, 1∶250, Abcam; rabbit Ki67; 1∶500 Abcam; rabbit IGF2 1∶400, Cell Signaling); rabbit anti-Wls, 1∶2000, gift from Richard Lang; mouse β-catenin 1∶100 BD Biosciences) were used for indirect immunofluorescence and immunohistochemistry. All control/mutant pairs were photographed at the same magnification. Number of Msx2^+^ cells was counted from a fixed field in 10 different sections from 4 embryos.

Proliferation index was assessed by percent of cells with Ki67 expression in the Runx2 expression domain, in the dermal mesenchyme in the Twist2 domain, and surface ectoderm in the Keratin14 expressing cells. Similar numbers of cells in each domain were analyzed between four controls and mutants. Statistical significance for all quantifications was calculated using two-tailed Student *t*-test.

### Alcian blue and Alizarin red and AP staining

Embryos were sacrificed, skinned and eviscerated, fixed in 95% ethanol, then stained for 24 hours each in 0.03% Alcian blue and 0.005% Alizarin red. Stained embryos were subsequently cleared in graded series of potassium hydroxide and glycerol until photography, after which they were stored in 0.02% Sodium Azide in glycerol. Whole mount Alkaline phosphatase staining was performed as previously described [63] with the addition of a 70% ethanol overnight incubation step after fixation in 4% PFA.

### RT-PCR

Cranial mesenchyme and surface ectoderm were micro-dissected from E12.5 embryos and flash frozen in liquid nitrogen. Total RNA was isolated using the Qiagen RNEasy micro kit, and cDNA was reverse transcribed using the ABI kit. RT-PCR for most of the Wnt ligands was amplified for 35 cycles of 94°C for 15 seconds, 66°C for 30 seconds, and 72°C for 60 seconds and the products were resolved on a 3% agarose gel. For Wnt1, 5b, 8a, 8b, 10b the annealing temperature was 55°C for 30 seconds. Primer sequences for RT-PCR are listed in Table 1.

## Supporting Information

Figure S1 Expression of Wnt ligands in total cranial ectoderm and mesenchyme. (A) RT-PCR for individual Wnt ligands was performed on cDNA from purified mouse embryonic cranial mesenchyme and surface ectoderm. (B) –RT negative control.
(EPS)

Figure S2 Later deletion of *Wls* in the ectoderm is dispensible for cranial bone ossification. (A,B) Whole-mount skeletal preps of embryonic mouse heads. P, parietal bone, f, frontal bone, n, nasal bone, ey, eye, mx, maxilla. Scale bar represents 5 mm.
(EPS)

Figure S3 Mesenchymal deletion of *Wntless* with Engrailed1Cre leads to diminished bone differentiation. (A–D) Whole-mount skeletal preps of embryonic mouse heads. P, parietal bone, f, frontal bone, n, nasal bone, ey, eye, mx, maxilla. Scale bar represents 5 mm. Indirect immunofluorescence with DAPI-stained (blue) nuclei (G, K, M, Q) or in situ hybridization (H, I, N, O) was performed on E13.5 coronal embryonic head sections. β-galactosidase staining (E, F, E′, F′), Von Kossa staining (J, P), or alcian blue staining (L, R) was performed on coronal embryonic head sections and counterstained with eosin at the indicated stages. fb, forebrain, mn, meningeal progenitors, vhf, supraorbital vibrissae hair follicle. Green arrowheads indicate meningeal progenitors, black arrowheads indicate hair follicles, and red arrow demarcates the dorsal extent of ossified frontal bone. High magnification images (E′, F′) with accompanying low magnification and box depicting inset (E, F). Control and mutant panels are shown at the same magnification and scale bars represent 100 µm.
(EPS)

Figure S4 Deletion of ectoderm *Wntless* does not compromise cell survival and ectodermal differentiation. Indirect immunofluorescence with DAPI-stained (blue) nuclei (A–F), in situ hybridization (G, H), β-gal staining (K) was performed on coronal embryonic head sections. E12.5 embryonic heads were stained in whole mount for AP activity to detect bone primordia (black arrow in I, J). Note that in the *Creect; Wls^fl/fl^* mutant, the frontal bone rudiment is not detectable (red arrows in J). Inset in A, shows positive control for active caspase 3 immunostaining in the developing eye. Diagram of embryonic head in (A) inset depicts region of interest and plane of section. Boxed areas correspond to high magnification panels (E, F, E′, F′) and white-hatched lines demarcate the surface ectoderm (E′, F′). Fb, frontal bone; pb, parietal bone, cs coronal suture.
(EPS)

Figure S5 Deletion of ectoderm *Wntless* leads to decrease in cell survival of brachial arch mesenchyme but not patterning. In situ hybridization of various facial mesenchyme patterning markers (A–H) and indirect immunofluorescence of activate caspase 3 with DAPI stained nuclei to identify dying cells (I, J) was performed on coronal E12.5 head sections. Diagram of embryonic head in (A) inset depicts region of interest and plane of section.
(EPS)

Figure S6 Deletion of mesenchyme *Wntless* does not compromise cell survival, ectoderm differentiation, and proliferation. Indirect immunofluorescence with DAPI stained nuclei (A–D). Percentage of Ki67+ proliferating cells in the osteoprogenitors, dermal progenitors and surface ectoderm at E12.5 and E13.5 (E). Boxed areas correspond to high magnification panels (C′, D′).
(EPS)

Figure S7 Cranial dermal and osteoprogenitors are distinct lineages during embryonic development. Indirect immunofluorescence with DAPI stained nuclei (A–C). Boxed areas correspond to high magnification panels (A′–C′).
(EPS)
